# A Novel Supplementation Approach to Enhance Host Response to Sublingual Vaccination

**DOI:** 10.1038/s41598-018-36370-8

**Published:** 2019-01-24

**Authors:** John C. Rowe, Zayed Attia, Eunsoo Kim, Estelle Cormet-Boyaka, Prosper N. Boyaka

**Affiliations:** 1 Department of Veterinary Biosciences, The Ohio State University, Columbus, OH USA; 2 Infectious Disease Institute, The Ohio State University, Columbus, OH USA

## Abstract

Sublingual immunization is emerging as an alternative to nasal immunization and induction of mucosal IgA responses. Using *Bacillus anthracis* edema toxin (EdTx) as an adjuvant, we previously showed that innate responses triggered after sublingual immunization could limit generation of IgA responses. We tested whether co-administration of a neutrophil elastase inhibitor (NEI) could rescue the ability of EdTx to induce broad antibody responses, including mucosal IgA. NEI supplementation of sublingual vaccines containing EdTx promoted antigen-specific serum IgA responses but also enhanced serum IgG1, and IgG2b responses. This enhancing effect of NEI did not extend to all antibody isotypes and IgG sublclasses, since NEI  reduced serum IgE responses and did not affect IgG2a/c and IgG3 responses. NEI supplementation also promoted anti-*Bacillus anthracis* protective antigen (PA) neutralizing antibodies and enhanced high affinity IgG1 and IgA antibodies. In addition to serum IgA, NEI supplementation stimulated antigen-specific mucosal IgA responses in the GI tract, and enhanced antigen-specific IgG responses in vaginal washes. Analysis of CD4^+^ T helper cell responses revealed that co-administration of NEI broadened the profile of cytokine responses, by stimulating Th1, Th2, Th17, and Tfh cytokines. We also noted that NEI had a higher stimulatory effect on IL-5, IL-10, IL-17 responses.

## Introduction

Needle-free vaccines delivered via mucosal surface have the potential of being better-accepted by the most vulnerable and commonly vaccinated population of children. They also present higher likelihood to generate the necessary B and T cell responses for optimal protection at the portal of entry of infectious agents, in addition to promoting the levels of systemic immunity generally achieved by conventional injected vaccines^1^. Secretory IgA (SIgA) represents the hallmark of immune responses at mucosal surfaces. The high resistance of these polymeric immunoglobulins to degradation in the harsh environment of mucosal surfaces, including the lumen of the gastrointestinal tract, allow them to provide frontline protection at the portal of entry of most infectious microbes^2^. While mucosally delivered subunit-vaccines have the potential of stimulating broad mucosal and systemic immune responses, their ability to trigger mucosal IgA relies on the addition of effective vaccine adjuvants. Stimulation of inductive site immune responses in different mucosal sites (i.e., gastrointestinal tract, respiratory tract, rectal) imprints the expression of discrete mucosal homing receptors and adressins which allow effector B and T cells to home in distinct mucosal tissues. For example, intranasal delivery of vaccines containing appropriate mucosal adjuvants can promote specific immune responses in the airways. However, safety issues were reported following intranasal application of  a ganglioside-binding toxin as adjuvant. Sublingual immunization is now being considered as an alternative to the intranasal route of vaccination. Nonetheless, a major challenge for the development of sublingual vaccines is the identification of appropriate antigen-adjuvant formulations^2,3^.

We have previously shown that *Bacillus anthracis* edema toxin (EdTx) is an effective adjuvant capable of promoting both systemic immunity and mucosal SIgA responses against nasally co-administered vaccine antigens^4,5^. However, when EdTx was tested as adjuvant for sublingual vaccination, it promoted antigen-specific IgG responses in the bloodstream but failed to elicit IgA responses in the serum or mucosal secretions^6^. This lack of IgA responses was not due to the route of immunization itself, since sublingual immunization could induce these responses when vaccines were administered with a range of adjuvants including bacterial enterotoxins, toll-like receptor ligands, and STING ligands^7–11^. Interestingly, the lack of IgA response correlated with the recruitment of neutrophils after sublingual administration of EdTx, and partial depletion of neutrophils before sublingual immunization restored the adjuvant activity of EdTx for IgA responses^6^. Neutrophils represent the largest population of myeloid cells in the bloodstream and characterize the initial response to inflammatory events through their own degranulation and cytokine production^12^. Unfortunately, depletion of neutrophils prior to immunization is not a feasible approach and thus, new strategies are needed to improve the efficacy of EdTx-based, and possibly other, sublingual vaccines.

Neutrophils are recruited by inflammatory cytokines, including IL-6, IL-1β, and TNFα, and were more recently recognized to contribute to the chemotaxis of other myeloid cells through the products released after neutrophil degranulation^12,13^. Neutrophil elastase inhibitors (NEI) are a class of serine protease inhibitors that target the neutrophil granule protein elastase, commonly implicated in chronic lung inflammation^14–16^.

## Results

### Co-administration of a NEI enhances serum IgG1 and IgG2b responses to a sublingual vaccine

**Figure 1:**
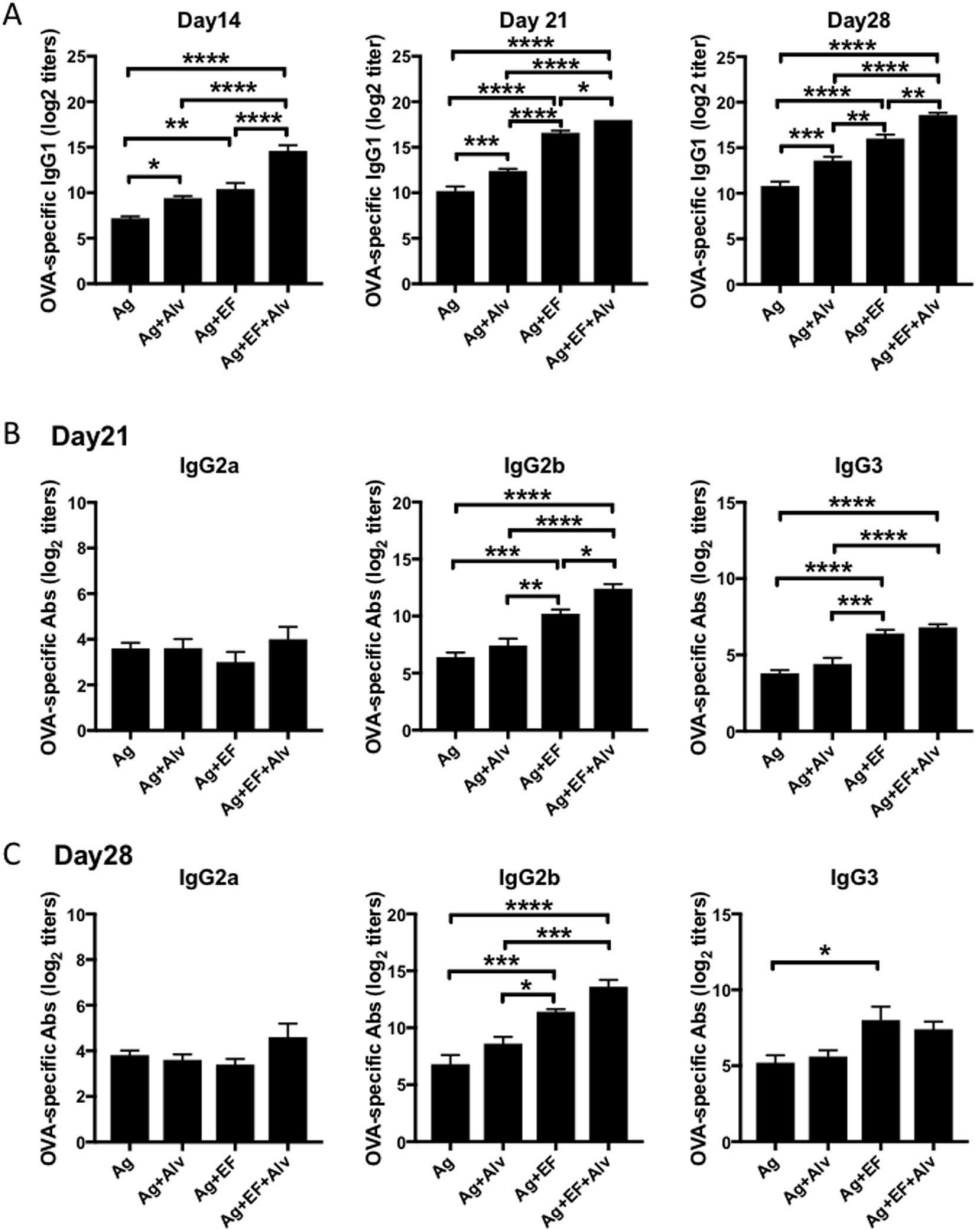
Sublingual co-application of a NEI enhances antigen-specific serum IgG1 and IgG2b responses. Mice were immunized three times at weekly intervals by sublingual application of antigens (OVA plus PA) alone or antigens plus the NEI Alvelestat. To assess the effect of NEI supplementation on the adjuvanticity of *Bacillus anthracis* edema toxin [EdTx, which results from combination of PA plus edema factor (EF)], mice were immunized with antigens plus EF or antigens plus EF plus NEI. Serum samples were collected weekly and OVA-specific Ab responses were analyzed by ELISA. (**A**) Kinetic of IgG1 Ab responses. (**B**,**C**) IgG2a/c, IgG2b and IgG3 Ab responses were assessed: (**B**) one week (Day 21), or (**C**) two weeks (Day 28) after the last immunization. Data are expressed as mean Ab titers ± SD (n = 5 mice per group) and are from three independent experiments. *p ≤ 0.05; **p ≤ 0.01; ***p ≤ 0.001; ****p ≤ 0.0001.

We first asked whether supplementation with a NEI could affects IgG responses induced by a model sublingual vaccine containing EdTx as adjuvant. Vaccines targeting two or more pathogens can reduce the schedule of vaccination. We choose to use Ovalbumin (OVA) plus Bacillus anthracis protective antigen (PA) as a combinatorial antigen to test the ability of NEI to regulate the immune response to two different antigens. OVA is a well-studied model antigen which allow a more in-depth analysis of immune responses to vaccination thanks to the large number of reagents available to study OVA-specific B and T cell responses. On the other hand, the use of PA as antigen allowed us to address the biological significance of the antibody responses through the assessment of anti-PA antibodies (Abs) ability to neutralize anthrax lethal toxin (LeTx). Analysis of OVA-specific IgG1 responses revealed that NEI increased the magnitude of responses induced by EdTx, and this effect was evident as early as day 14 after the first immunization (Fig. 1A). We also found that the NEI used in these studies had an intrinsic adjuvant activity and increased IgG1 responses when co-administered with antigen in the absence of EdTx (Fig. 1A). Analysis of other IgG subclasses showed no evidence that the NEI enhanced IgG2a/c, or IgG3 when co-administered with antigen in the absence of EdTx (Fig. 1B,C). However, the NEI significantly increased serum IgG2b responses induced by EdTx as adjuvant (Fig. 1B,C).

### Supplementation with a NEI suppresses serum IgE responses induced by a sublingual vaccine

**Figure 2:**
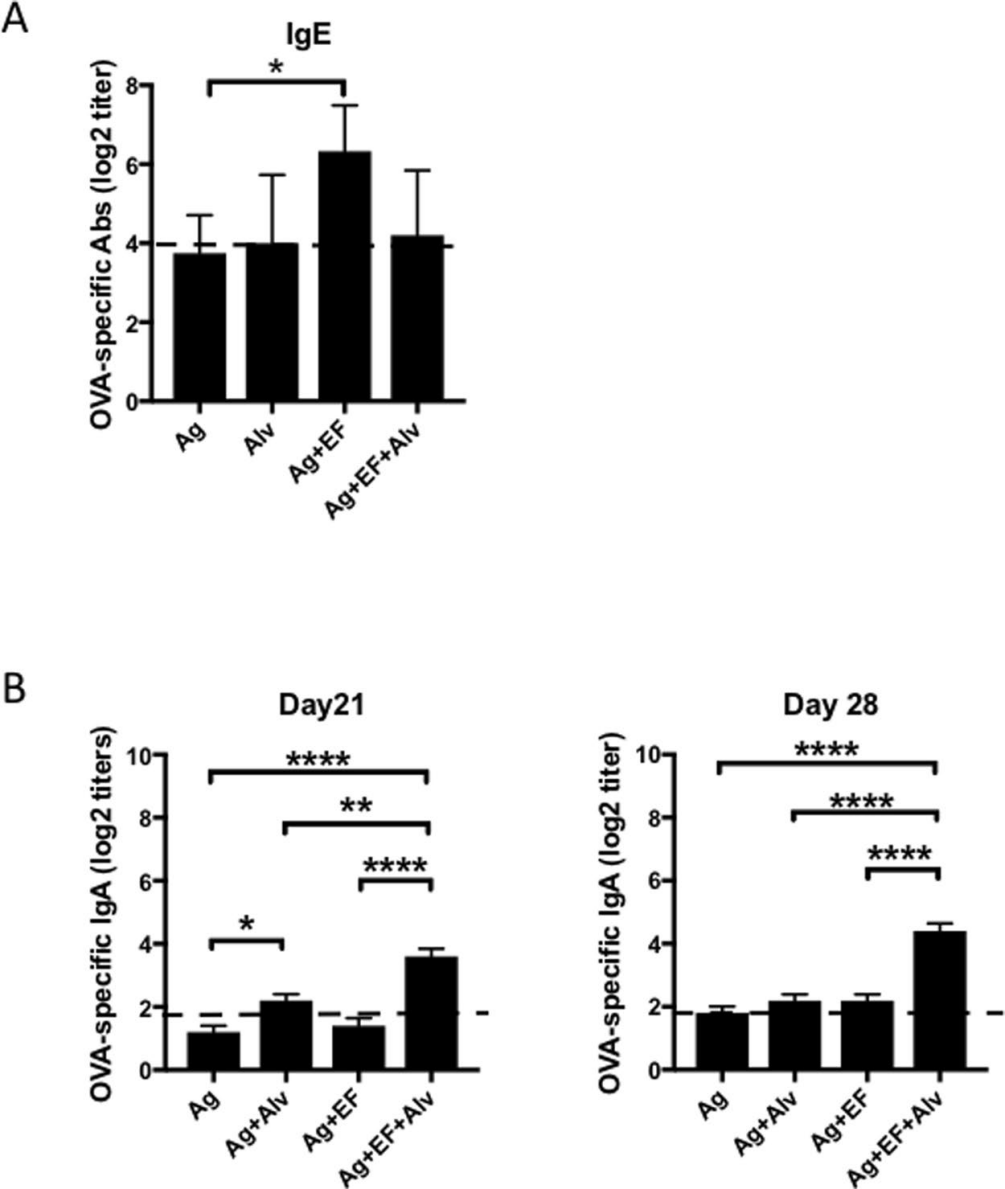
Sublingual co-application of a NEI differentially affects antigen-specific serum IgE and IgA responses. Mice were immunized three times at weekly intervals by sublingual application of antigens (OVA plus PA) alone, antigens plus the NEI, antigens plus EF, or antigens plus EF plus NEI. Serum samples were collected weekly and OVA-specific Ab responses were analyzed by ELISA. (**A**) OVA-specific IgE responses on day 14. (**B**) OVA-specific IgA responses on days 21 and 28. Data are expressed as mean Ab titers ± SD (n = 5 mice per group) and are from three independent experiments. *p ≤ 0.05; **p ≤ 0.01; ***p ≤ 0.001; ****p ≤ 0.0001.

Since the presence of NEI differentially regulated IgG subclass responses, we next addressed its potential effects on other immunoglobulin isotypes. Sublingual immunization with EdTx as adjuvant induced OVA-specific IgE responses which peaked 2 weeks after the first immunization (day 14) (Fig. 2A). Co-administration of NEI significantly reduced the magnitude of IgE responses to levels barely at the detection threshold (Fig. 2A). Furthermore, no IgE response was detected following sublingual application of NEI and antigen alone (Fig. 2A).

### Supplementation of a sublingual vaccine with a NEI promotes serum IgA responses

As previously reported^6^, sublingual immunization with EdTx as adjuvant failed to induce OVA-specific serum IgA responses (Fig. 2B). Mice co-administrated antigen and NEI showed detectable but minimum levels of serum IgA responses at day 21. Minimum serum IgA levels were also induced and could be measured on day 28 in mice immunized with antigen (OVA + PA) plus EF. Interestingly, only mice immunized with antigen and EF in the presence of the NEI developed significant serum IgA responses (Fig. 2B).

### NEI supplementation enhances the amount of high affinity Abs and toxin neutralizing responses in mice immunized with EdTx as adjuvant

**Figure 3:**
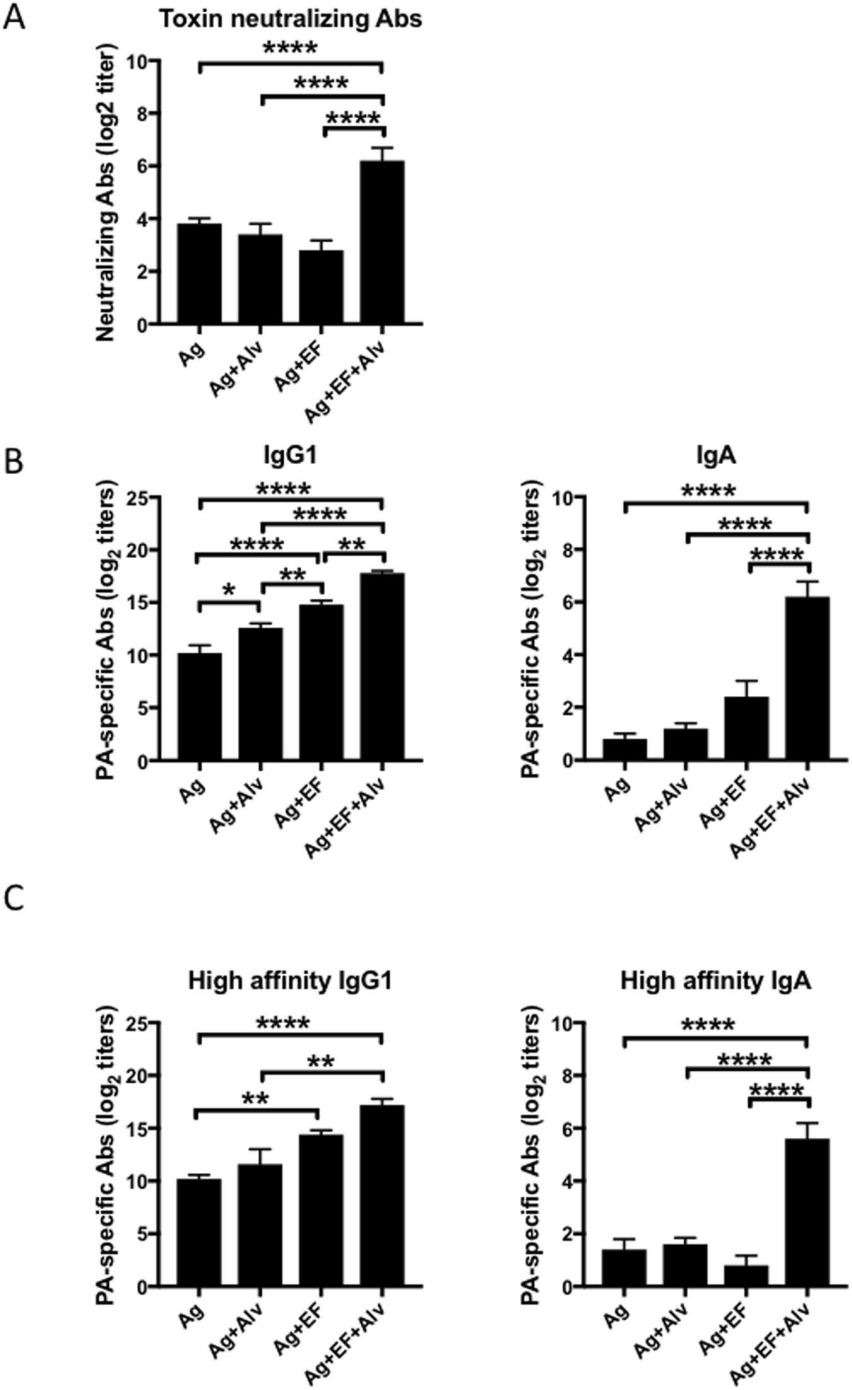
Sublingual co-application of a NEI promotes high affinity Abs with enhanced neutralizing activity. Mice were immunized three times at weekly intervals by sublingual application of antigens (OVA plus PA) alone, antigens plus the NEI, antigens plus EF, or antigens plus EF plus NEI. (**A**) Serum anti-PA neutralizing Ab responses. Serum samples were collected one week after the last immunization (Day 21), and PA-specific neutralizing Ab titers were determined by the *in vitro* macrophage toxicity assay. (**B**) PA-specific IgG1 and IgA Ab responses were determined by classical ELISA. (**C**) High affinity PA-specific IgG1 and IgA Ab responses. Antibody titers were assessed by a modified ELISA where low affinity Abs were removed by incubation with urea. Data are expressed as mean Ab titers ± SD (n = 5 mice per group) and are from three independent experiments. *p ≤ 0.05; **p ≤ 0.01; ***p ≤ 0.001; ****p ≤ 0.0001.

Results summarized above show that NEI supplementation improves the kinetic of IgG1 responses and allows mice immunized with EdTx to reach maximum IgG1 titers more quickly (Fig. 1). They also show that NEI broadens the antibody responses by enhancing IgG2b and promoting IgA (Figs 1 and 2). To determine the biological significance of the broader antibody responses measured in the blood of mice administered vaccines supplemented with NEI, we first assess anti-PA neutralizing antibodies. Serum of mice immunized with antigen alone (OVA plus PA), antigen plus NEI, or antigen plus NEI contained low and comparable levels of anti-PA neutralizing antibodies (Fig. 3A). On the other hand, anti-PA neutralizing antibody titers were significantly increased in the group of mice that received EdTx and NEI (Fig. 3A).

We also addressed whether the enhanced neutralization of LeTx toxicity was due to the increase in anti-PA antibody titers or whether it also resulted from an increase in high affinity antibodies. As with anti-OVA responses above (Fig. 1), NEI supplementation enhanced the titers of PA-specific IgG1 and IgA responses in mice with EdTx as adjuvant (Fig. 3B). Most importantly, the IgG1 and IgA promoted by NEI supplementation were high affinity antibodies (Fig. 3C).

### Supplementation of a sublingual vaccine with a NEI promotes mucosal IgA responses

**Figure 4:**
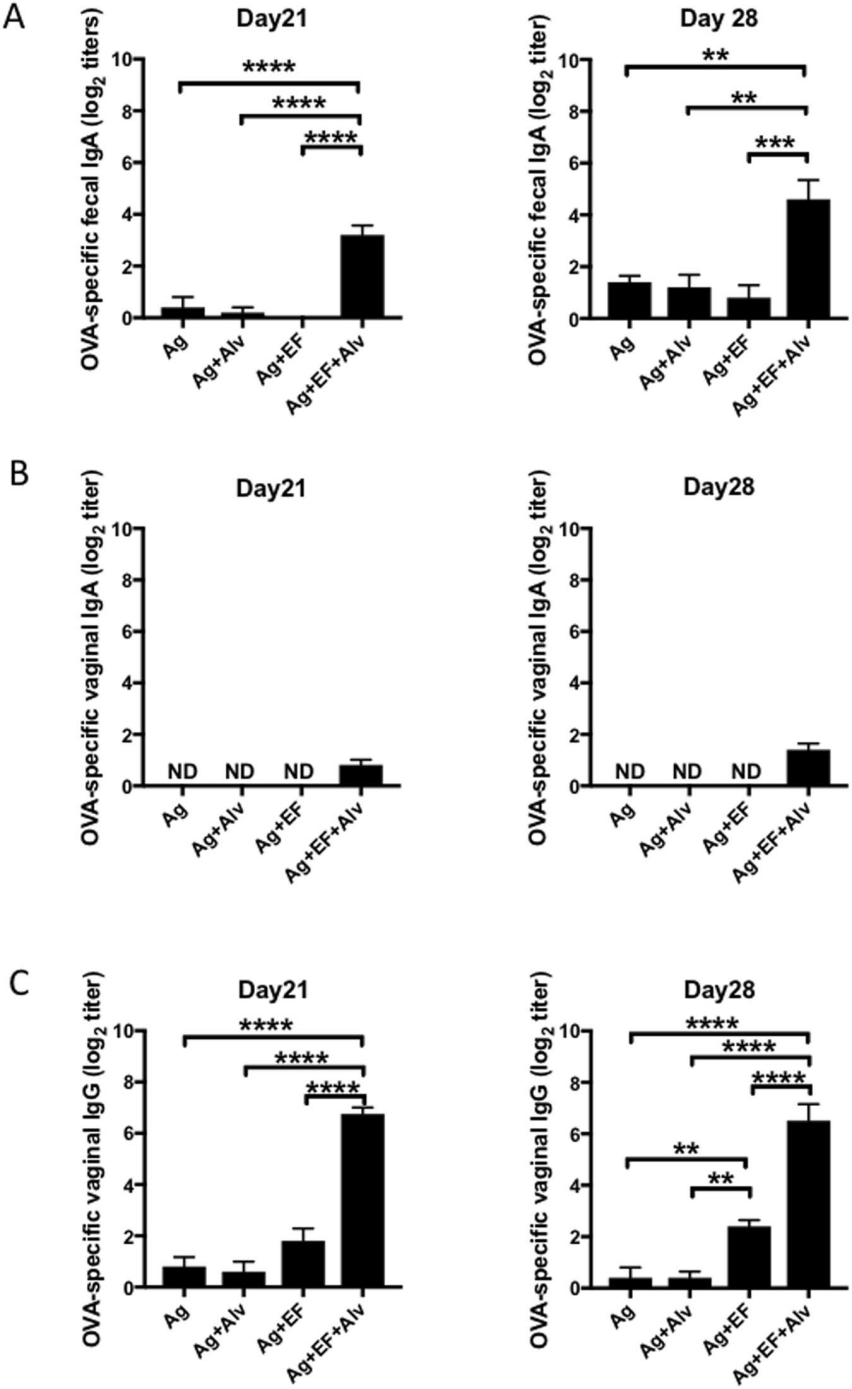
Sublingual co-application of a NEI promotes fecal IgA responses. Mice were immunized three times at weekly intervals by sublingual application of antigens (OVA plus PA) alone, antigens plus the NEI, antigens plus EF, or antigens plus EF plus NEI. Fecal pellets and vaginal washes were collected weekly and OVA-specific Ab responses were analyzed by ELISA. (**B**) OVA-specific IgA responses in fecal extracts on days 21 and 28. (**B**) OVA-specific IgA responses in vaginal washes on days 21 and 28. (**C**) OVA-specific IgG responses in vaginal washes on days 21 and 28. Data are expressed as mean Ab titers ± SD (n = 5 mice per group) and are from three independent experiments. *p ≤ 0.05; **p ≤ 0.01; ***p ≤ 0.001; ****p ≤ 0.0001.

Our previous studies have shown that a major limitation of EdTx as adjuvant for sublingual vaccines is its inability to promote IgA responses in the bloodstream and in mucosal secretions^6^. Sublingual application of antigen alone or antigen plus NEI did not stimulate antigen-specific IgA responses in the gut. Addition of NEI to a vaccine formulation containing antigen and EdTx resulted in the induction of fecal IgA responses, which were detectable 3 weeks after the first immunization (day 21) and were further increased one week later (day 28) (Fig. 4A). To determine if NEI supplementation promoted generalized IgA responses in other mucosal secretion, we analyzed these responses in vaginal washes. Unlike mucosal secretions of the gastrointestinal  tract, the vaginal washes of mice immunized with EdTx and NEI showed only non-significant levels of antigen-specific IgA responses (Fig. 4B). However, NEI supplementation was able to enhance the vaginal IgG responses induced by EdTx as adjuvant (Fig. 4C). These data suggest that NEI might promote expression of gut homing receptors by IgA producing cells, while the elevated IgG antibodies found in the secretions of the genitourinary tract might be coming from diffusion of IgG from the blood. It is important to indicate that anti-PA Ab responses measured in mucosal secretions of mice which  received the NEI were of the same profile and magnitude than anti-OVA Ab responses (not shown).

### Addition of NEI to a sublingual vaccine broadens the profile of CD4^+^ T helper cell responses

**Figure 5:**
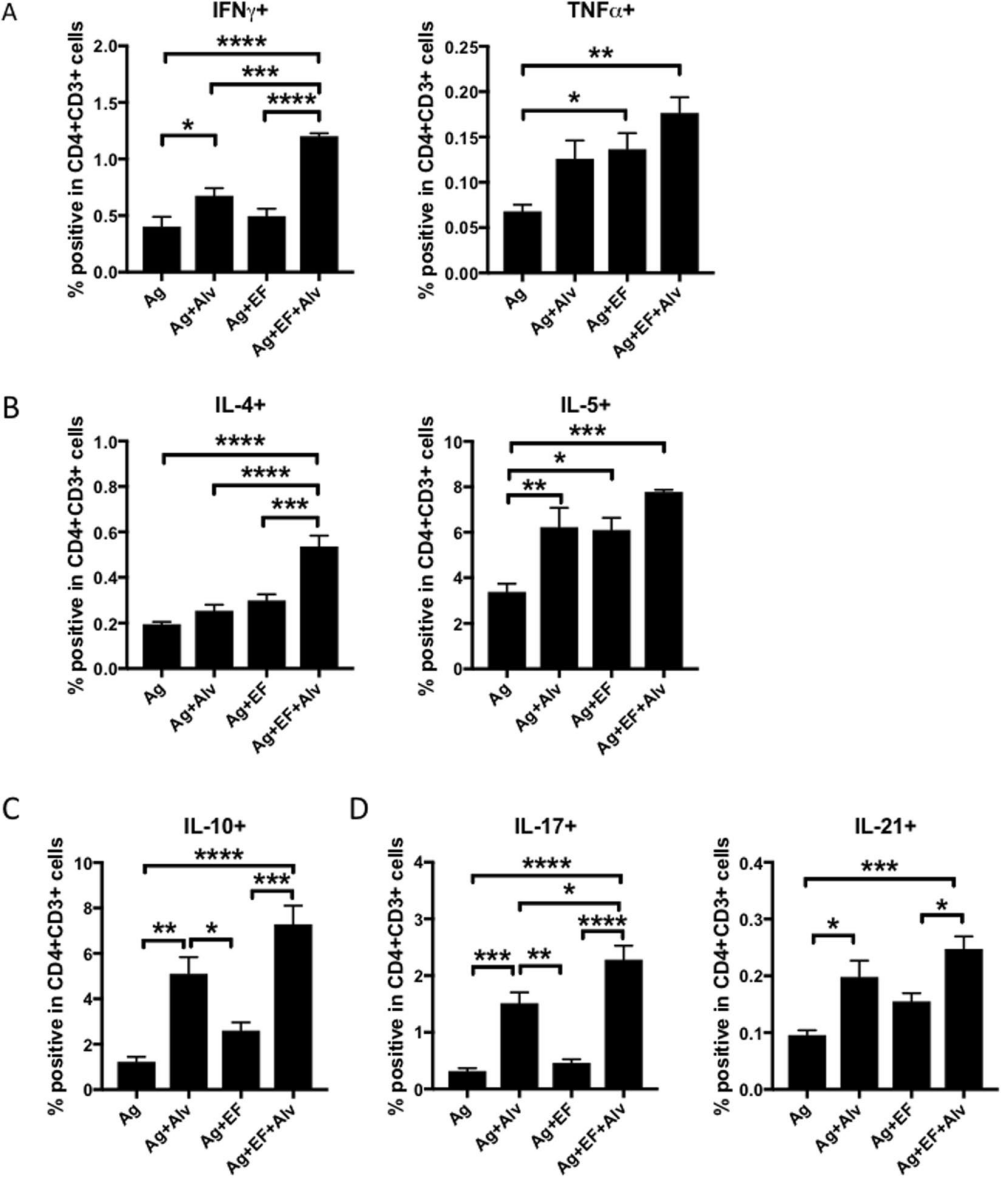
Addition of NEI to a sublingual vaccine broaden the profile of CD4^+^ T helper cell responses. Mice were immunized three times at weekly intervals by sublingual application of antigens (OVA plus PA) alone, antigens plus the NEI, antigens plus EF, or antigens plus EF plus NEI. Spleen were collected two weeks after the last immunization and single cell suspensions were cultured for 5 days in the presence of OVA (1 mg/ml). Frequencies of (**A**) CD4^+^ Th1 cells; (**B**) CD4^+^ Th2 cells; (**C**) CD4^+^ Th17 andCD4^+^ Tfh cells were then analyzed by flow cytometry. Data are expressed as mean ± SD (n = 5 mice per group). *p ≤ 0.05; **p ≤ 0.01; ***p ≤ 0.001; ****p ≤ 0.0001.

We performed flow cytometry analysis of cytokines produced by CD4^+^ T cells upon *in vitro* restimulation with OVA to elucidate the profile of T helper cytokines that supported the antibody responses induced by supplementation with NEI. Spleen cells from mice immunized with antigens plus NEI exhibited the same frequencies of Th1 cells (IFNγ^+^CD4^+^ T cells and TNFα^+^CD4^+^ T cells) as spleen cells from mice that received antigen plus EdTx (Fig. 5A). The frequencies of Th2 cells producing IL-5 were also similar between these two groups, but mice immunized with antigens plus NEI exhibited lower frequencies of IL-4^+^ CD4^+^ T cells (Fig. 5B). These analyses also show that immunization with antigen plus NEI promoted higher frequencies of IL-10^+^ CD4^+^ T cells and IL-17^+^ CD4^+^ T cells than immunization with antigen plus EdTx (Fig. 5C,D). Furthermore, NEI and EdTx promoted the same frequencies of antigen-specific IL-21^+^CD4^+^ T cells (Fig. 5D). Consistent with the broad increase in antibody responses, co-administration of EdTx and NEI significantly increased all CD4^+^ T helper cytokine responses, including Th1, Th2, Th17 and Tfh responses (Fig. 5).

## Discussion

The sublingual route is currently used for drug delivery and allergen specific immunotherapy (ASIT). Sublingual vaccination is theoretically very simple as it involves application of liquid or rapidly dissolving tablets to the floor of the oral cavity. Like vaccines delivered at other mucosal sites, sublingual vaccines have the potential of producing mucosal SIgA in addition to IgG responses in the general bloodstream. Because sublingually administered vaccines are less likely to reach central nervous system tissues, they are potentially safer than nasal vaccines. Despite these advantages, identification of appropriate adjuvant formulations remains a requirement for the development of sublingual vaccines. Here we show that supplementation of the EdTx adjuvant with a chemical inhibitor of a neutrophil function broadens B and T cell responses induced after sublingual immunization and promotes antigen-specific SIgA Abs.

We have shown that the breath of antibody responses induced by EdTx as a sublingual adjuvant is limited by the recruitment of neutrophils in mucosal inductive sites^6^. While this work was the first to establish an inverse relationship between the presence of neutrophils and production of IgA, the mechanisms underlying the suppressive effect of neutrophils on IgA production remained elusive. It was previously shown that neutrophil peptide defensins enhance IgG, but not IgA responses against nasally co-administered vaccine antigen^17^. The primary (or azurophilic) granules of neutrophils contain defensins, myeloperoxidase, lysozymes, and three serine proteases: neutrophil elastase, cathepsin G and protease 3^12,18^. We now show that inhibition of a single function of neutrophils can rescue the adjuvant activity of EdTx and promote IgA responses both in the blood stream and in mucosal tissues. This finding is significant because the use of a serine protease inhibitor might be a more realistic alternative to neutrophil depletion as a strategy for promoting IgA responses. It also raises the question of the mechanism behind neutrophil elastase limiting IgA production. Neutrophil elastase and the related serine proteases cathepsin G and proteinase 3, were shown to regulate cytokine responses through activation or degradation of cytokines, cytokine receptors, or toll-like receptor^12,19^. Our separate studies have shown that NEI induce the expression of IL-10 mRNA but also AID and BAFF responses by spleen cells *in vitro*^20^. Because IL-10 facilitates Ig class switching for production of IgA^21^ and regulate the expression of AID for induction of Ig CSR^22^, this may be a mechanism for enhanced IgA responses in mice sublingually immunized with EdTx in the presence of NEI.

Sublingual immunization of mice with STING ligands as adjuvant induces IgA responses in mucosal tissues of the airways^7,10^ and the genitourinary tract^10^. However, neither CpG nor the STING ligand 3′33-cGAMP as sublingual adjuvant induced SIgA in the secretions of the GI tract (i.e., in fecal extracts)^10^. We now show that when added to an EdTx-based sublingual vaccine, NEI primarily promoted SIgA responses in mucosal secretions of the GI tract. No significant levels of IgA were measured in other mucosal secretions although IgG responses were significantly increased in vaginal washes. The best-described factors that regulate homing of immune cells into the gut are CCR9 and gut homing receptors α4β7^23,24^. These factors are induced through signals provided by retinoid acid, a product of myeloid cells in mucosal tissues^23^. To our knowledge, no study has examined whether neutrophils regulate expression of gut homing receptors. But it is worth noting that in conditions of normal homeostasis, neutrophils are not present in mucosal tissues where the concentration of IgA is the highest in the body. The observation that NEI primarily promotes SIgA in the gut suggests that it either directly stimulated retinoic acid production or stimulated cytokines such as IL-5, IL-6 and IL-21, which promote or enhance expression of gut homing receptors^25,26^.

IgA was not the only immunoglobulin isotype affected by supplementation of the EdTx-based sublingual vaccine with NEI. In fact, NEI also enhanced IgG1 and IgG2b responses. This is consistent with the previous report that elastase inhibits the maturation and function of dendritic cells, including expression of the costimulatory molecules CD40, CD80 and CD86^27^. Thus, the presence of NEI may have promoted the maturation of dendritic cells and their expression of BAFF and APRIL, and perhaps costimulatory molecules (i.e., CD40), for enhanced antibody production. Another important point revealed in the present study is that the presence of NEI improved the quality of antibody responses. Indeed, NEI increased the titers of high affinity IgG and IgA antibodies, an effect that translated into higher titers of anti-PA neutralizing antibodies. Our findings are in line with the recent report that neutrophils regulate germinal center formation in secondary lymphoid organs and production of autoreactive plasma cells in lupus^28^. Analysis of antigen-specific T helper cytokine responses provided some clues on how NEI improved the breath, the magnitude and the quality of antibody responses in mice immunized via the sublingual route with EdTx as adjuvant. We found that addition of NEI resulted in higher frequencies of Th1 cells producing IFN-γ, Th2 cells producing IL-5 and IL-10, Th17 cells producing IL-17, and Tfh cells producing IL-21. This broad profile of Th cell cytokine responses has most likely contributed to increase B cell survival via signals from IL-5 and IL-17^29–33^. It is also consistent with the observation by others that neutrophils preferentially accumulate in proximity of T cells in secondary lymphoid organs during the early stage of lupus and that neutrophil depletion accelerate germinal center formation^28^. In our study, the NEI may have played the same role as neutrophil depletion and enhanced development of germinal centers and production of high affinity antibodies by facilitating differentiation of Tfh producing IL-21.

Altogether, our results show that supplementation of sublingual vaccine by co-administration of a NEI can improve the kinetic, breath and quality of antibody responses both in the bloodstream and in mucosal tissues. These findings raise the question of can NEI display similar adjuvant activity for vaccines given through other mucosal routes and, possibly non-mucosal vaccines? Our separate studies indicate that this is in fact the case and that co-administration of NEI modulates immune responses induced by intranasal and injected vaccines (Attia Z and Boyaka PN, *manuscript in preparation*). It is also worth noting that NEI alone exhibited some adjuvant activity and enhanced IgG1 and IgA responses against the sublingually co-administered vaccine antigens. This effect does not appear to extend to other immunoglobulin isotypes/subclasses. Previous studies have established that after oral administration, the plasma concentration of the NEI Alvelestat peaked by 05–1.5 hrs and that it was eliminated thereafter^34^. Thus, the NEI is believed to act quickly after sublingual immunization and to be eliminated within 24 hrs. Future studies will investigate the underlying mechanisms and whether NEI can be used as a universal strategy for improving immune responses to sublingual vaccines regardless of the adjuvant or delivery system.

## Material and Methods

### Ethics statement

All experiments were performed according to the guidelines for the Care and Use of Laboratory Animals adopted by the National Institutes of Health. The Institutional Animal Care and Use Committee (IACUC) at The Ohio State University approved all protocols. All efforts were made to avoid unnecessary pain and distress and to minimize animal suffering during the course of these studies.

### Animals

Female C57BL/6J mice were obtained from The Jackson Laboratory (Bar Harbor, ME). Mice were maintained in a specific pathogen-free environment and used at 10 ± 2wks of age. All the experiments were approved by and performed in accordance with the guidelines of NIH and The Ohio State University IACUC.

### Sublingual immunization and samples collection

Sublingual immunization was performed as previously described^6,10^ by administration of 10–13 μl of a PBS solution to mice anesthetized by intraperitoneal injection of ketamine/xylazine (0.1 ml of a 20 mg/ml ketamine/20 gm body weight). Each anesthetized mouse received antigen [i.e., 20 μg of PA (*Bacillus anthracis* protective antigen, BEI Resources, Manassas, VA) plus 40 μg of ovalbumin grade V (Sigma-Aldrich, Saint Louis, MO)] in PBS. To assess the adjuvant activity of *Bacillus anthracis* edema toxin [which results from combination of PA and *Bacillus anthracis* edema factor (EF), groups of mice were given antigens plus 15 μg of EF (BEI Biosource)]. To address the effect of NEI supplementation, groups of mice were given antigens plus 100 μM of Alvelestat (AZD9668, C_25_H_22_F_3_N_5_O_4_S) (Selleckchem, Houston, TX), or antigen plus EF plus Alvelestat. Mice that received the NEI showed no change in their vitality, food consumption or  body weight. All groups were immunized at weekly intervals for 3 consecutive weeks (days 0, 7, and 14). Serum, fecal pellet and vaginal wash samples were collected weekly, and nasal washes were collected at day 28.

### Evaluation of antigen-specific antibody responses

To determine OVA-specific and PA-specific antibody titers, ELISA were performed with antigen-coated plates as described previously^4–6,35,36^ Briefly, microtiter plates were coated with OVA (1 mg/ml) or PA (15 μg/ml). For detection of OVA- or PA-specific IgG and IgA Abs, serum or fecal material extracts were serially diluted in PBS 1% BSA, added to the plates and the binding antibodies were detected with HRP-conjugated anti-mouse γ- or α-heavy chain-specific antisera (Southern Biotech Associates Inc., Birmingham, AL). Biotin-conjugated rat anti-mouse IgG1, IgG2a/c, IgG2b or IgG3 monoclonal Abs and HRP-conjugated streptavidin (BD Bioscience, san Jose, CA) were used to measure IgG subclass responses. The reactions were revealed by addition of the water-soluble HRP substrate ABTS (2,2′-Azinobis [3-ethylbenzothiazoline-6-sulfonic acid]-diammonium salt, Sigma-Aldrich) and the Ab titers were determined as the last dilutions of samples with an absorbance of >0.1 above that of control samples from naïve mice.

For assessment of IgA levels in the intestinal secretions, freshly emitted fecal pellets were normalized by homogenization in PBS (1 ml per 0.1 g feces). After centrifugation, dilutions of supernatants were used for evaluation of antigen-specific IgA levels as described above.

### Quantification of high affinity antibody responses

High affinity antibody responses were measured by ELISA as described above with a minor modification. Briefly, plates were coated with PA and incubated with dilutions of the samples. Urea (4 mM) was then added and the plates incubated for 30 min at room temperature to remove antibodies that bound to the antigen with low affinity^37^. After washing, detection antibodies were added and the remaining steps of the ELISA conducted as described above.

### Assessment of toxin neutralizing antibodies

Toxin neutralization assay was performed as previously described^4–6^. Briefly, sample dilutions were added to J774 macrophages cultured in cultured in RPMI supplemented with 10% fetal calf serum. *Bacillus anthracis* lethal toxin (LeTx) [i.e., PA plus *Bacillus anthracis* lethal factor (LF, List Biological, Campbell, CA)] was then added to the plates. After overnight incubation, MTT (3-(4,5-dimethylthiazol-2-yl)−2,5-diphenyl tetrazolium bromide; Sigma-Aldrich) was added to assess the viability of macrophages as a function of redox potential. Toxin neutralizing antibody titers were determined as the lowest concentrations of sera  that protect macrophages from the cytotoxicity of LeTx.

### Analysis of antigen-specific T helper cell cytokines

Antigen-specific T helper cell cytokine responses were analyzed by flow cytometry after *in vitro* restimulation and intracellular staining with cytokine-specific fluorescent antibodies. Briefly, splenocytes and cervical lymph nodes were collected on day 28 after the first immunization and restimulated with antigen (i.e., OVA) *in vitro* as previously described^4–6^. After 5 days culture, cells were subjected to extracellular staining with lineage-specific antibodies [i.e., anti-CD3, and anti-CD4 (Biolegend, San Diego, CA)] and, after fixation, to intracellular staining with Th1, Th2, Th17, and Tfh cytokine-specific antibodies (Biolegend). Labeled cells were then analyzed with an Attune NxT flow cytometer (Thermo Fisher Scientific, Waltham, MA).

### Statistical analysis

Results are expressed as mean ±one standard deviation. Statistical significance was determined by one-way ANOVA, followed by Tukey post-hoc test. All statistical analyses were performed with the StataSE 12.0 software (StataCorp LLC, College Station, TX) and Prism 7 software (Graphpad Software, La Jolla, CA).
